# Bumblebees distinguish floral scent patterns, and can transfer these to corresponding visual patterns

**DOI:** 10.1098/rspb.2018.0661

**Published:** 2018-06-13

**Authors:** David A. Lawson, Lars Chittka, Heather M. Whitney, Sean A. Rands

**Affiliations:** 1 School of Biological Sciences, University of Bristol, Bristol BS8 1TQ, UK; 2 Department of Experimental and Biological Psychology, Queen Mary University of London, London E1 4NS, UK

**Keywords:** crossmodal learning, floral volatiles, multimodal signal, olfaction, plant–pollinator interaction, sensory modality

## Abstract

Flowers act as multisensory billboards to pollinators by using a range of sensory modalities such as visual patterns and scents. Different floral organs release differing compositions and quantities of the volatiles contributing to floral scent, suggesting that scent may be patterned within flowers. Early experiments suggested that pollinators can distinguish between the scents of differing floral regions, but little is known about how these potential scent patterns might influence pollinators. We show that bumblebees can learn different spatial patterns of the same scent, and that they are better at learning to distinguish between flowers when the scent pattern corresponds to a matching visual pattern. Surprisingly, once bees have learnt the spatial arrangement of a scent pattern, they subsequently prefer to visit novel unscented flowers that have an identical arrangement of visual marks, suggesting that multimodal floral signals may exploit the mechanisms by which learnt information is stored by the bee.

## 1. Introduction

Flowers act as multisensory billboards [1], guiding their pollinators using visual patterns [2], heat [3,4], electrical interactions [5], tactile surfaces [6], humidity patterns [7] and scent [8]. Floral scents are composed of a huge variety of differing volatile compounds [9], and different organs within the same flower have been shown to release differing compositions and quantities of these volatiles [10–22], suggesting that pollinators may experience patterns of scent when visiting a plant. Less work has been done in exploring the microstructure of these patterns within individual flowers, but there is evidence that there are differences in volatile production across the surface of individual petals in *Nicotiana suaveolens* [23], *Stephanotis floribunda* [23], *Mirabilis jalapa* [24], *Ranunculus acris* [11], *Linaria vulgaris* [25] and *Melampyrum pratense* [25]. This suggests that there could be subtle scent signals that allow a visiting pollinator to orientate itself on or within the flower [11]. Early experiments [18,19] showed that pollinators may be able to distinguish between the scents of differing regions of the same flower, and solitary bees *Chelostoma rapunculi* alter their response to differently scented organs of *Campanula trachelium* once they have experienced a rewarding flower [22]. However, little is known of the effects that potential patterns of scent have upon the behaviour of a pollinator visiting the flower, where the scent patterns on a petal may act as nectar guides. Given that visual nectar guides are demonstrably important for enhancing pollinator efficiency [26,27], it is therefore possible that pollinators may respond to patterns of scent in a similar manner. Here, we describe an experiment that demonstrates that bumblebees can learn to distinguish between flowers with differing patterns of the same scent.

Potentially, scent patterns alone could enhance interactions between pollinators and flowers. However, it is more likely that these patterns will be combined with signals over other sensory modalities. Multimodal signals have been shown repeatedly to help pollinators to learn floral signals [28–30], improving their foraging efficiency, and benefiting plants by increasing pollinator floral constancy [31]. These multimodal signals often occur in patterns that overlay each other, such as pigment patterns corresponding with patterns of tactile surface structure [32]. Given that olfactory signals are easy to learn and remember and enhance recognition speed in combination with visual signals [33], combining a floral scent with other signal modalities is particularly effective [34]. This has also been shown in moths [35], and it is known that bimodal signals that include an olfactory element enhance learning and discrimination in bumblebees *Bombus terrestris* [36], ants *Cataglyphis fortis* [37] and fruit flies *Drosophila melanogaster* [38]. It is therefore likely that scent patterns may correspond to visual patterns within a flower, such as visual nectar guides, which on their own are known to enhance interactions with pollinators [26,39]. Furthermore, scent cues and visual cues enhance each other when one or the other is rendered ambiguous by the environment [31,40], and scent patterning may therefore be particularly important for plants growing in highly variable light environments [41]. Here, we investigate the interaction between scent and visual patterns, and demonstrate that learning in bumblebees is enhanced when patterns overlap. We also demonstrate that bumblebees are able to transfer pattern information learnt in one sensory modality (scent) to a differing novel modality (visual) without additional learning, suggesting that multimodal floral signals may exploit the mechanisms by which learnt information is stored by the bee.

## 2. Methods

### (a) Flight arena and bumblebee colony conditions

Flower-naive *B. terrestris* colonies (Koppert BV, Berkel en Rodenrijs, Netherlands and Syngenta-Bioline, Little Clacton, UK) were connected to a flight arena via a transparent gated tube which could be manually manipulated to regulate which bees, and how many, could enter or leave the arena—see [42,43] for full details of the arena, light conditions and animal husbandry. Bees were fed 30% sucrose solution daily *ad libitum* after experiments had taken place and pollen was added directly to the colony three days a week. Foraging individuals were marked on their thorax with an identifying pattern of non-toxic bee-marking paint (E. H. Thorne, Rand, UK). For all the experiments described, we used marked foragers that had not previously been used and that were naive to the scent and visual stimuli described, but which had experience of drinking from a variety of artificial flowers of different designs to those used here.

### (b) Scent pattern learning and test of transfer to visual pattern, using sucrose in rewarding and quinine in non-rewarding aversive artificial flowers

#### (i) Training

**Figure 1.**
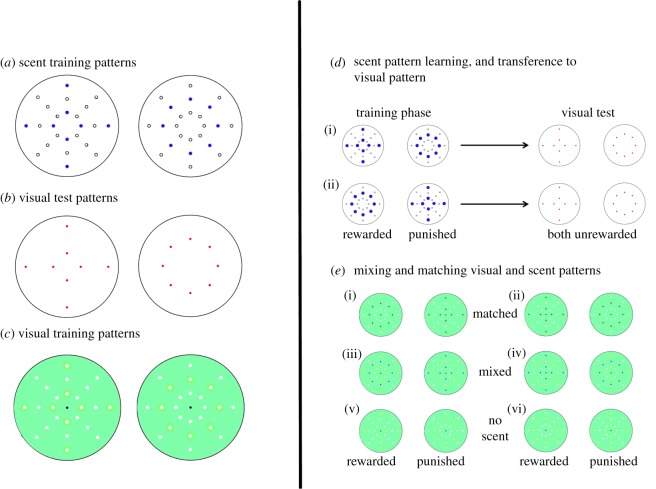
Patterns presented, and outline of the learning regimes. (*a*) Scent training patterns, where empty circles denote unscented wells containing mineral oil, and filled blue circles denote scented wells containing the diluted peppermint oil. (*b*) Visual marker test patterns. (*c*) Visual training patterns: note that eight of the holes on each disc are lined with a green circle that subtly differs in shade to the background—the positioning of these visual stimulus circles correspond to the eight wells on each flower in (*a*) that received a scent stimulus. The visual discs were placed on top of the discs containing filled wells, with holes (white circles) aligned with the wells. In (*a*–*c*), a drinking cup was placed in the centre of the topmost disc. (*d*) Sketch of the scent pattern learning trials, and the corresponding visual marker test (presented in figures 2 and 3), where the cross (i) or circle (ii) was the rewarding stimulus. (Note that the spots denoting scent positions have been enlarged for clarity, and do not represent any physical enlargement in the experimental apparatus.) (*e*) Sketch of the multimodal stimulus learning tests (presented in figure 4), where either the scent and visual patterns corresponded so that scented wells were marked (i and ii), or where the rewarded scented pattern corresponded to the non-rewarded visual pattern and *vice versa* (iii and iv), or where bees learnt a visual pattern without a corresponding scent pattern (v and vi). See also table 1 for details of training regimes (i–vi).

Bees were individually trained to differentiate between patterns on artificial flowers using differential conditioning. The artificial flower stimuli were created from white Perspex discs (75 mm diameter, 3 mm thick). Each disc had 24 holes (2 mm diameter) drilled in them, following the pattern shown in figure 1*a*, and the upturned lid of a 0.5 ml Eppendorf container was glued to the centre of each disc as a drinking cup. At the start of each session, the bottom of each disc was covered with a fresh layer of self-adhesive covering film, causing the drilled holes to become wells capable of containing a droplet of liquid. The adhesive covering film was removed from the discs at the end of each day and the discs were left to soak overnight in a detergent solution to remove volatiles and glue. Discs then received either a ‘cross’ or ‘circle’ scent pattern, where the eight wells indicated in figure 1 received 2.5 µl of peppermint solution (a 1 : 10 mix of peppermint oil : mineral oil, with peppermint oil supplied by Amphora Aromatics, Bristol, UK; peppermint oil has previously been demonstrated to be learned by *B. terrestris* in association with sugar in scent experiments [31]). In order to eliminate any potential visual cue resulting from scented oil droplets being patterned in the flower, the other 16 unfilled wells received 2.5 µl of pure (non-scented) mineral oil.

**Table 1.** Details of the disc scent and visual patterns for the six training regimes used in experiment 2, with details of the number of bees trained (*n*) for each regime. In each training period, five of the rewarding and five of the non-rewarding disc types were used in the arena. Regimes correspond to the patterns described in figure 1*e*.

regime	discs with rewarding stimulus	discs with aversive stimulus	*n*
i	visual circle, scented circle	visual cross, scented cross	10
ii	visual cross, scented cross	visual circle, scented circle	10
iii	visual cross, scented circle	visual circle, scented cross	7
iv	visual circle, scented cross	visual cross, scented circle	8
v	visual cross, no scent pattern	visual circle, no scent pattern	10
vi	visual circle, no scent pattern	visual cross, no scent pattern	10

To conduct the differential conditioning training, we simultaneously presented each bee with five ‘cross’-scented discs and five ‘circle’-scented discs. All flowers were presented as horizontal surfaces. In each training session, the central drinking cups of one group of discs received a reward stimulus of 30% sucrose solution (20 µl), while the drinking cups of the other group received a non-rewarding aversive stimulus of 0.12% quinine hemisulphate salt solution in water (20 µl): it has previously been demonstrated that bumblebees are unable to discriminate between the two solutions prior to landing [44]. Within an experimental training period, either crosses or circles consistently contained the reward, while the other group consistently contained the aversive stimulus, meaning that individual bees were trained to recognize a single consistent pattern of either a scented cross or a scented circle as a rewarding stimulus.

At the beginning of a training phase, the flight arena was cleared of bees and the gated tube connecting to the nest was blocked. The two groups of scent-patterned discs were then placed on top of transparent Sterilin containers (60 mm height) and distributed randomly throughout the flight arena. The forager was then allowed entry into the flight arena. The sequence of landings on rewarding discs and non-rewarding discs was noted, along with whether the forager also drank from the central well. Foragers interspersed their visits to the discs with returns to their nest. On exiting the arena to return to the nest, the arena was temporarily barred so that the stimuli discs could be swabbed with ethanol to remove scent marks and then placed into a new random arrangement to avoid foragers learning the spatial location of rewarding discs. Any discs that had been depleted from were replenished with sucrose solution.

We assumed that a bee had satisfactorily learnt to discriminate between scent patterns when it had landed and drunk at least ten times (not counting any landings where no drinking occurred), with at least eight out of ten consecutive drinking events being on rewarding discs. Here, we use the verb ‘to drink’ to describe any behaviour where the bee touched the contents of the drinking-cup with its mouthparts, and could therefore include probing behaviour where none of the stimulus was drunk. Twenty individuals reached these criteria with circles of scent as their rewarding stimulus, and 21 individuals reached these criteria with rewarding crosses (training was initiated in 67 bees, of which these 41 individuals reached the defined criteria). From the data collected for these individuals, we quantified a bee's learning speed as the cumulative number of landings (including those where the bee does not drink) up to and including the number of landings at which the bee drinks, and at least eight out of the ten previous consecutive drinks (including the current drink) are on a positive stimulus. The landing number metric was compared between the two different patterns using a Welch two-sample *t*-test, where the logarithm of the landing number metric was used to satisfy test assumptions. All statistical analyses were conducted using R [45], and all data described in the paper are available in the electronic supplementary material.

#### (ii) Visual marker test

To test whether bees were able to transfer their pattern learning between different sensory modalities, we challenged trained bees with a novel visual stimulus that corresponded to the learnt spatial scent pattern. Circular visual stimulus discs were created with white paper (75 mm diameter): five were marked with red points corresponding to the circular scent placement pattern used in the training phase, and five were marked with the cross pattern (figure 1*b*). The discs were then covered with self-adhesive covering film, to both allow them to be cleaned and to mask any potential scent from the ink spots. Each disc had an upturned Eppendorf lid (0.5 ml) glued to its centre, and each disc was attached to a white plastic disc (75 mm diameter, 3 mm thick) for stability.

Bees were tested immediately after they had completed their training. We imposed an additional training criterion that the individual had landed consecutively on at least 40 discs (where these 40 landings extended over multiple bouts, and could be on rewarding or non-rewarding discs). This reduced our test cohort of trained individuals to 33 individuals (16 with circles as their rewarding stimulus, 17 with crosses). As soon as a bee had reached these training criteria and had then returned to its nest, it was temporarily barred from the arena. The scent stimulus discs were removed, and the two sets of visual stimulus discs were then placed on 60 mm high Sterilin containers and arranged randomly throughout the flight arena with sucrose in the Eppendorf lid cups. The trained bee was then allowed to enter the flight arena, and its first ten landings were recorded before wiping the discs clean with ethanol. Of the 33 individual foragers that had reached training criteria, 30 landed on the visual stimuli at least ten times (of which 15 were trained to crosses as a rewarded stimulus, and 15 trained to circles). The number of visits (out of the first ten landings) that each of these individuals made to the cross pattern was recorded.

An additional control group of 16 bees were tested for their spontaneous preference for the visual stimuli. These naive individuals had experience of drinking from Eppendorf lid cups, but did not undergo any of the scent pattern training, and had not experienced the visual stimuli prior to testing. At the beginning of the test phase, they were released into an arena containing the two sets of five visual stimuli described above, and the number of visual cross stimuli they visited during their initial ten visits was recorded. Because these control animals had experienced no prior training, the Eppendorf cups contained 20 µl of sucrose solution to encourage visiting. Once a control individual had completed at least ten landings and returned to its nest, the discs were swabbed with ethanol to remove any scent marks.

The number of visits to visual cross stimuli were compared between bees trained with the two odour pattern stimuli and the untrained controls using a Kruskal–Wallis test (correcting for tied ranks) as the data did not fit requirements for parametric testing. *Post hoc* pairwise comparison of the three categories was conducted using nonparametric multiple comparisons with tied ranks [46]. To explore whether there were any biases by the untrained control bees for either pattern, we compared the number of initial landings on crosses and circles between both the controls and the two regimes of trained bees, using a two-tailed Fisher's exact test on the resulting 3 × 2 contingency table. We also tested whether there was a correlation between the number of visits it took a trained bee to learn its task (using the learning statistic defined above) and the number of visits it made (during its first ten visits) to the visual stimulus that corresponded to the pattern of its training scent stimulus. Correlations were explored using Spearman rank correlation tests as the data did not fit requirements for parametric testing.

### (c) Scent pattern learning and test of transfer to visual pattern, using sucrose in rewarding and water in non-rewarding artificial flowers

Using quinine as an aversive stimulus is a fast and effective technique for the differential conditioning of bees (e.g. [5,6,32,44]), but may not represent a natural task, as very few flowers encountered in the wild will present a distasteful nectar. We therefore conducted an additional set of experiments where bees instead encountered non-rewarding flowers during the training phase that contained water rather than quinine. Using water as a non-rewarding stimulus could mean that bees learn more slowly than with an aversive stimulus, and may not retain a learnt association as effectively. Furthermore, although it is unlikely that the bees could detect the difference between quinine and sugar solution prior to tasting it [44], using water also allows us to discount the bees avoiding quinine through any olfactory or visual cues that we had failed to detect ourselves.

Bees were trained as described previously, but with water as a non-rewarding stimulus, rather than quinine: 15 were trained to rewarded crosses, and 16 to rewarded circles. An additional probe test was added at the end of the learning period to confirm that the bees had learnt the patterns that were presented. Once a bee had reached the learning criteria, it was presented with a new set of flowers which had the same number of cross pattern and circle pattern flowers as the training phase (five cross pattern flowers and five circle pattern flowers). Both flower types used in this extra testing phase had water in their central drinking cups, meaning there was no rewarding pattern. The bee was allowed to visit twenty flowers (recording whether the bee attempted to drink from the flower on each of the visits), before being temporarily isolated under a 60 mm Sterilin container. After emptying the arena of flowers, the bee was then allowed to return to the nest before the next test phase.

The proportion of times an individual bee drank on a ‘trained rewarding’ and on a ‘trained non-rewarding’ flower were compared for bees trained to the two differing positive rewards with a linear mixed model using lme4 1.1 [47], considering the interaction between the training type and pattern visited, and including bee identity as a random term. A restricted maximum-likelihood approach was used following recommendations by [48], and significance tests were calculated for the model using Satterthwaite approximations for degrees of freedom, using lmerTest 2.0 [49].

Before bees were tested for their ability to transfer scent patterns to visual stimuli, they experienced additional retraining to counteract any change in behaviour in response to the unrewarded learning probe test. During this retraining phase, the bees were given two or three foraging bouts where they presented with the same rewarding and non-rewarding discs as in their original training phase. After this retraining, the bees then experienced the visual marker test as described previously, except that all the central drinking cups contained water rather than sucrose solution.

Analyses were conducted as described for the previous experiment. For the additional unrewarded scent pattern test phase, we calculated the proportion of landings that led to drinking behaviour when the bee landed on either the originally rewarded pattern or the originally unrewarded pattern. These two sets of proportions were compared with a Wilcoxon test, as the data could not be transformed to satisfy assumptions of normally distributed data.

### (d) Differential conditioning to a spatial fragrance pattern and visual pattern combination

An additional experiment was conducted to test whether matching patterning in different sensory modalities could aid learning of rewarding patterns. Each bee tested was presented with ten stimuli discs similar to those described in the previous experiments. Each disc additionally had one of two patterns printed on a transparent plastic film placed on top of it (where the printed side had an additional layer of transparent self-adhesive covering film to preserve the printed pattern). Both patterns had 24 holes (1 mm diameter) aligned with the 24 wells on the plastic discs to allow exposure to volatiles. Both patterns (figure 1*c*) consisted of a green background of hue 140° HSB (with saturation 50% and brightness 100%). Eight of the 24 holes were surrounded with green circles (10 mm diameter) of 120° HSB. Green patterning was chosen rather than colouring that more closely resembled a bee-pollinated flower, in order to make the bee engage thoroughly with the artificial flower. Previous experiments have shown that bees are able to discriminate between these two shades of green [5,30,31,39,40], but the task is sufficiently difficult to ensure that the bees do not simply fly straight to the nectar source [50] without engaging with the flower. For five discs these 1 mm diameter circles were in a cross pattern (‘visual cross’), and the remaining five discs had these circles in a circular pattern (‘visual circle’). The upturned lid of a 0.5 ml Eppendorf container was glued to the centre of each of these plastic film discs as a drinking cup. As well as presenting different visual patterns, the two sets of discs were treated with scent patterns as described in figure 1*a*, receiving either a crossed scent pattern, a circular scent pattern, or no mineral oil or scent (as a control). Combining scent and visual patterns, our experiment consisted of six sets of training regimes described in table 1 and figure 1*e*.

Naive bees (that had not been used for the first experiment) were trained in an identical manner to the training phase of the previous experiments, but with each bee consistently experiencing one of the regimes of rewarded and aversive patterns described in table 1, using sucrose or quinine solutions as described previously. We assumed that a bee had learnt to discriminate between scent patterns when it had landed and drunk at least ten times (not counting any landings where no drinking occurred), with at least eight out of ten consecutive drinks being on rewarding discs. We extracted the same summary statistic measuring speed of learning as described earlier. In total, 55 bees were trained (numbers are given in table 1) and analysed.

We compared the measure of learning using a two-way ANOVA design (considering scent pattern, visual pattern, and the interaction between the two), after taking log transforms of the data to ensure the test statistic assumptions were met. The interactions were significant, and were compared using a *post hoc* least-squares means test using lsmeans within R [51].

## 3. Results

### (a) Scent pattern learning and test of transfer to visual pattern

#### (i) Training

When trained using differential conditioning [44], bees learned to distinguish between the two different scent patterns. Bees that were trained to recognize crossed scent patterns as a rewarded stimulus showed no difference in learning time compared to those trained with rewarding circles (with quinine as the aversive stimulus—circle: 31.15 ± 2.78 (mean ± s.e.) visits (*n* = 20); cross: 24.19 ± 2.25 visits (*n* = 21); Welch's *t-*test, *t*_38.71_ = 2.01, *p* = 0.052; with water as the non-rewarding stimulus—circle: 49.00 ± 7.37 visits (*n* = 16); cross: 64.40 ± 7.64 visits (*n* = 15); Welch's *t-*test, *t*_27.76_ = 1.69, *p* = 0.103).

**Figure 2.**
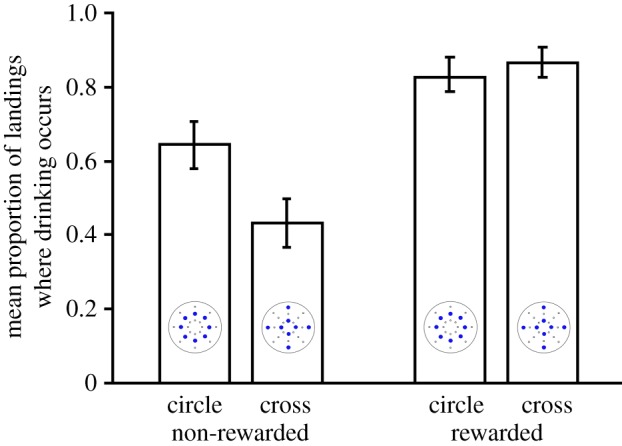
Bees attempted to drink more frequently when they landed on the pattern they had been trained to associate with a reward. Mean proportion of landings (±s.e.) during the non-rewarded probe phase which led to drinking, for bees that originally been trained to rewarding cross patterns and non-rewarding circle patterns, or *vice versa*. See results section for statistical analysis.

In the experiment where water was presented as the non-rewarding stimulus, in the intermediate test phase where bees trained with a non-rewarding water stimulus were challenged with scented but unrewarded patterns, the bees attempted to drink more frequently when they landed on the pattern they had been trained to associate with a reward (*F*_1,31_ = 61.63, *p* < 0.001; figure 2). This was influenced by whether the pattern was a circle or a cross, where bees trained to rewarding crosses showed greater discrimination between patterns (pattern alone: *F*_1,31_ = 1.70, *p* = 0.202; interaction between pattern and training: *F*_1,31_ = 9.94, *p* = 0.004; figure 2). Bees are therefore able to learn and distinguish between flower types differing in spatial arrangements of the same scent.

#### (ii) Visual marker test

**Figure 3.**
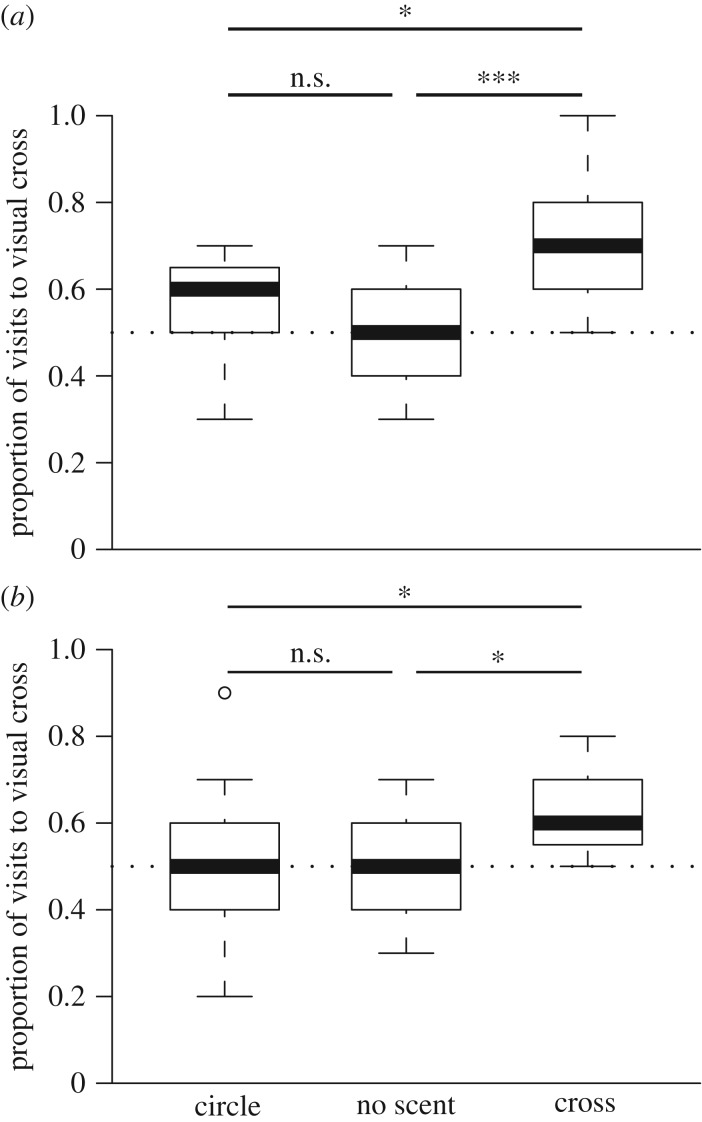
Bees trained to scented cross patterns are more likely to choose corresponding visual cross patterns when presented with novel visual stimuli. (*a*) Quinine-trained experiment. (*b*) Water-trained experiment. Boxplot shows the median and interquartile range (IQR), with whiskers showing the maximum value within 1.5 × IQR, and individual points mark values outside this range.

In the experiment where bees experienced an aversive quinine solution, bees visited the flowers with crosses differently between the treatments and the control (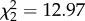, *p* = 0.002; figure 3*a*). *Post hoc* comparisons demonstrated that the bees trained to cross-shaped scent patterns were more likely to visit visual crosses than either the scent-naive control bees (*p* = 0.001) or bees trained to a circular scent pattern (*p* = 0.038), but there was no difference between the control and circle-trained bees (*p* = 0.24). There was no difference in the initial choices made by either the control or trained bees (Fisher exact: *p* = 0.140).

In the experiment in which non-rewarding flowers contained water, bees also visited the crosses differently between the treatments and the control (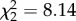, *p* = 0.017; figure 3*b*). *Post hoc* comparisons demonstrated that bees trained to cross-shaped scent patterns were more likely to visit visual crosses than were scent-naive control bees (*p* = 0.036) or bees trained to a circular pattern (*p* = 0.029), but bees trained to circular scent patterns did not visit crosses differently to control (*p* = 0.915) bees. There was no difference in the initial choices made by either the control or trained bees (Fisher exact: *p* = 0.171).

There was no relationship between the number of visits needed to learn a task and the number of visits a bee made to the visual stimulus that corresponded to its trained scent stimulus (quinine-trained experiment: *r_s_* = −0.21, *S* = 5440, *n* = 30, *p* = 0.265; water-trained experiment: *r_s_* = 0.11, *S* = 4421, *n* = 31, *p* = 0.561).

### (b) Differential conditioning to a spatial fragrance pattern and visual pattern combination

**Figure 4.**
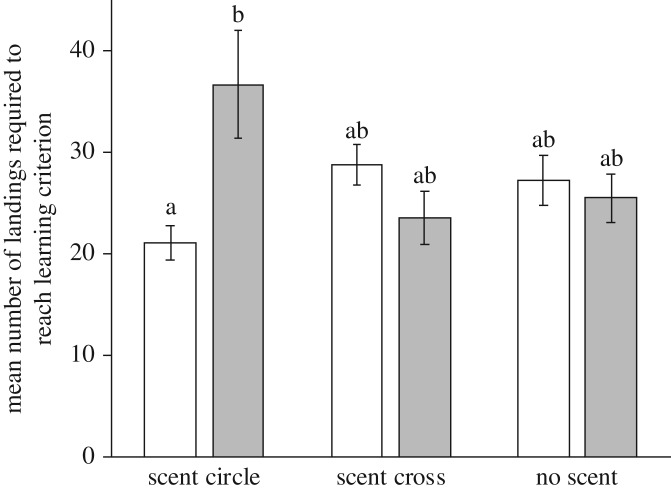
Bees learn multimodal stimuli in fewer visits when the visual and scent patterns match, when compared with mixed patterns that are in conflict with each other. Plot shows mean number of landings required (±s.e.) to reach the learning criteria for training flowers showing the three types of scent pattern and either visual circles (unfilled white bars) or visual crosses (filled grey bars). Separation of interaction means were compared using a least-squares test, and letters denote means that did not differ (*p* ≥ 0.05).

When considered separately, neither the visual pattern (*F*_1,49_ = 0.39, *p* = 0.526) nor the scent pattern (*F*_2,49_ = 0.04, *p* = 0.964) had any influence on the number of visits required to reach the learning criterion, but the interaction between scent and visual patterns was important (*F*_2,49_ = 6.58, *p* = 0.003, figure 4), demonstrating that bees were faster at learning to identify a rewarding stimulus when the visual and scent patterns experienced were identical in their spatial layout, and slower when the visual and scent pattern did not match. *Post hoc* comparisons of the means for the interactions (figure 4) suggest that the bees were learning the flowers with circular scent patterns but differing visual patterns at different speeds, where the mismatched patterns appear to have been more difficult to learn.

## 4. Discussion

### (a) Bees can learn scent patterns

Our results suggest that floral scent patterns could be an effective discriminatory aid for pollinators on their approach to the nectaries. Our results show that bees are able to learn and distinguish between flower types' differing spatial arrangements of the same scent (figure 2), confirming the results suggested in [30]. The flowers of a wide phylogenetic range of species are known to show differences in the quantity and diversity of floral volatiles produced by different parts of the flower (including different areas of the petals, and other regions that the pollinators are interacting with when they are in close contact with the flower) [11–13,18–25], and it is therefore possible that these patterns may enhance the interaction between the pollinator and plant. Our experiments only considered a single uniform scent, demonstrating that the spatial pattern of a scent is sufficient to help guide a bee, and that further experiments could explore the subtleties of guiding that the multiple different scents within real flowers might provide. Patterning within a flower with localized concentration of scent may also act to alter the amount of scent required, fine-tuning the trade-offs seen between attracting pollinators and attracting or repelling herbivores [52]. Similarly, demonstrating that pollinators can distinguish between patterns of the same scent suggests that patterning is another filtering layer that a cheating mimic needs to overcome, enhancing the robustness of scent as an honest signal [33].

### (b) Bees can learn matched multimodal patterns faster

Our experiment found that bees could learn to identify flowers that combined similar multisensory patterns faster than flowers that had non-overlapping multisensory patterns (figure 4). Multimodal signals with matching scent and visual patterns therefore enhance learning, but the speed of learning is reduced when the spatial arrangement of the scent and visual patterns are incongruent. Therefore, matching patterns across different sensory modalities [32,43] may further enhance the learning benefits achieved with multimodality. This further increase in learning speed could occur because of the particular ways in which the bee's trajectory *en route* to the flower induces neural responses in both the visual and olfactory pathways (and indeed there could be other chemical properties of the floral surface that further induce these responses). Projection neurons from both the visual and olfactory sensory periphery converge onto the basal ring region in the mushroom bodies, prominent dorsal structures of the insect brain that mediate learning and memory and which function as coincidence detectors between rewards and other sensory input [53–55]. As the bee moves over a particular arrangement of visual and olfactory stimuli, a reproducible temporal pattern of neural activity in both visual and olfactory pathways will be generated that can be used to predict the reward. Since both these pathways project to a region of the mushroom bodies that is known to mediate learning and memory via synaptic plasticity [56], both signals may enhance the learning of each other when their spatial arrangements match.

### (c) Bees can transfer learnt patterns between sensory modalities

Our experiment shows that when bumblebees have learnt a scent pattern, they are then predisposed to the same pattern presented in a different sensory modality, without needing to experience the latter beforehand (figure 3). These findings are reminiscent of honeybees (*Apis mellifera*) learning a ‘sameness rule’ [57], where individuals are able to learn the concept of ‘sameness’ in the olfactory sensory modality and then apply it to novel visual stimuli. A cognitively loaded explanation of our findings might draw on similarities with the popular game where a person has to reach blindly into a bag, and identify various objects (e.g. a screw, or a clothes peg) by matching tactile information with previously stored visual information about shape. This would require the cognitive abstraction of shape beyond a particular pattern of neural activity that comes with the stimulus in one sensory modality. It is possible, for example, that bees sequentially explore the entire spatial arrangement of the scented wells within a pattern (in the same way as a human might run her fingers over an invisible object in a bag, to glean information about its shape), and in this way form a representation of a cross-shaped scented pattern or a circular one. Our inspection of the bees' behaviour during learning the scented patterns, however, indicates that such full sequential exploration of the scent patterns (e.g. by the bees tracing, in flight or by walking, the pattern of scented wells) looks like it does not occur, and a simpler explanation is therefore likely. Whatever the mechanisms are behind this sensory transfer, being able to transfer learnt information between sensory systems enhances interactions, and is thus of benefit to both pollinator and plant, and could drive the evolution of these interactions in highly variable environments where being able to switch between different sensory signals is important for accurately identifying a suitable flower.

## Supplementary Material

Datasets

## Data accessibility

This article has no additional data.
